# Supplementary material for: Magnitude, distribution and determinants of non-utilization of antenatal care services among women in low- and middle-income countries: Insights for implementation of WHO recommendations
Source: PLoS One. 2025 Aug 18;20(8):e0330596. doi: 10.1371/journal.pone.0330596 (PMC12360577; doi:10.1371/journal.pone.0330596)
Supplement: Attachment — Submitted filename: reviewers comments.pdf [file pone.0330596.s002.pdf]

Dear Editors and Authors,

Thank you for giving me the opportunity to review the manuscript, under the title “Magnitude, Geographic Distribution and Contributing Factors of Non-Utilization of Antenatal Care (ANC) Services among Women in Low- and Middle-Income Countries (LMICs): Insights for implementation of WHO recommendations”.

The authors chose a very important topic on maternal health care services in LMICs. This study examined data from DHSs (2015-2024) from 47 LMICs. The authors employed spatial analysis to show geographic distribution of ANC non-utilization and hierarchical analysis to identify factors associated with ANC non-utilization. The authors found that pooled magnitude of ANC non-utilization was at 10.59%, ranging 40.05% in Afghanistan to 0.76% in Burundi. Many regions in several countries identified as hotspots for ANC non-utilization. Factors significantly associated with higher odds of non-utilization were: no or low education, being unmarried, being in low wealth index, living in rural areas, distance to health facilities, and low-income levels of a country.

The manuscript is well written. The findings from this study have the potential to influence health interventions and policies to improve the use of ANC services in LMICs. The authors need to address the following issues before the manuscript can be considered for publication.

The full title seems a bit long. The authors may opt to use the short title and add few words to it.

In the abstract, the results should report the ORs (95%CI) for the factors significantly associated with non-utilization of ANC. If word count is an issue, the authors can summarize the prevalence of pooled non-utilization by stating for example, “Pooled prevalence of non-utilization of ANC among women in LMICs was at 10.59%, ranging from 40.05% in Afghanistan to 0.76% in Burundi.”, with many regions in several countries identified as hotspots for ANC non-utilization. Then, the authors can report the ORs for factors significantly associated with non-utilization of ANC.

Throughout the manuscript, the authors don’t need to write the full low- and middle-income countries. They just use it in full low- and middle-income countries (LMICs) when they use it first time in the manuscript; and then use the short term (LMICs) instead of the full term.

Under the sub-section “Magnitude of non-utilization of ANC services in LMICs”, the author should add in text that “Afghanistan had the highest non-utilization at 40.05% and Burundi had the lowest at 0.76%”.

For figure 4 (Hot spot analysis …) and figure 5 (Interpolation of non-utilization of ANC…), the authors should use a landscape of the maps as the current figures seem to be a bit confusing, showing the Asian countries (e.g., Tajikistan, Pakistan, India….) above Albania, Armenia, and Europe.
